# Prediction of renal outcome in Henoch–Schönlein nephritis based on biopsy findings

**DOI:** 10.1007/s00467-019-04415-3

**Published:** 2019-12-03

**Authors:** Mikael Koskela, Elisa Ylinen, Helena Autio-Harmainen, Heikki Tokola, Päivi Heikkilä, Jouko Lohi, Hannu Jalanko, Matti Nuutinen, Timo Jahnukainen

**Affiliations:** 1 Children’s Hospital, Pediatric Research Center, University of Helsinki, Helsinki University Hospital, Helsinki, Finland; 2 Department of Pediatric Nephrology and Transplantation, New Children’s Hospital, University of Helsinki and Helsinki University Hospital, PO Box 347, Stenbäckinkatu 9, 00029 Helsinki, Finland; 3 Medical Research Center Oulu and Department of Pathology, Oulu University Hospital, Oulu, Finland; 4 Department of Pathology, Helsinki University Hospital, Helsinki, Finland; 5 Department of Children and Adolescents, Oulu University Hospital, Oulu, Finland; 6 PEDEGO Research Unit, Research Unit for Pediatrics, Dermatology, Clinical Genetics, Obstetrics and Gynecology, Medical Research Center Oulu (MRC Oulu), Oulu, Finland

**Keywords:** α-SMA, Vimentin, PSGL-1, Semiquantitative, Oxford classification, Immunohistochemistry

## Abstract

**Background:**

In Henoch–Schönlein nephritis (HSN), a risk factor for unfavorable outcome is prolonged proteinuria, but the value of renal biopsies in prognosis assessment is debatable.

**Methods:**

We evaluated serial renal biopsies from 26 HSN patients. Follow-up biopsy occurred at median 2.1 years after diagnostic biopsy. Patients formed two groups at the follow-up biopsy: patients without proteinuria (group I; *n* = 11) and with proteinuria (group II; *n* = 15). Biopsies underwent evaluation according to three classifications: International Study of Kidney Disease in Children (ISKDC), Oxford (MEST-C), and semiquantitative classification (SQC) including an activity and chronicity score. Analysis also included expression of pro-fibrotic (alpha-smooth muscle actin and vimentin) and inflammatory (P-selectin glycoprotein ligand-1) molecules in the diagnostic biopsy specimens. Definition of unfavorable outcome was active renal disease or reduced renal function at last follow-up.

**Results:**

Between the biopsies, SQC chronicity score increased in 22 (85%) patients, whereas activity score and ISKDC grade decreased in 21 (81%) and 17 (65%), respectively. Of the MEST-C parameters, endocapillary proliferation (from 83 to 13%; *p* < 0.001) and crescents (from 63 to 25%; *p* = 0.022) showed significant reduction, and segmental glomerulosclerosis (from 38 to 79%; *p* = 0.006) significant increment. These changes occurred similarly in groups I and II. Expression of the pro-fibrotic and inflammatory molecules showed no clinically significant differences between groups I and II. None in group I and five (33%) patients in group II had unfavorable outcome (*p* = 0.053).

**Conclusions:**

Our results suggest that follow-up biopsies provide limited additional information to clinical symptoms in HSN outcome prediction.

**Electronic supplementary material:**

The online version of this article (10.1007/s00467-019-04415-3) contains supplementary material, which is available to authorized users.

## Introduction

The most common vasculitis in children is Henoch–Schönlein purpura (HSP) [1]. Its typical symptoms manifest in the skin, gastro-intestinal tract, joints, and kidneys, and the severity of the renal symptoms determines the long-term outcome. Of HSP patients, on average, 34% develop renal symptoms [2]. The symptoms are typically self-limiting proteinuria or hematuria, or both, but some patients develop severe proteinuria, and these patients are at greater risk for chronic kidney disease [3, 4].

For Henoch–Schönlein nephritis (HSN), the most common histologic classification is from the International Study of Kidney Disease in Children (ISKDC) [5]. Although it also considers mesangial proliferation, ISKDC classification is largely based on the percentage of glomeruli with crescents, and treatment modality selection is based on ISKDC grade. The prognostic value of the ISKDC classification is, however, questionable, giving rise to a need for a new classification [6, 7]. The Oxford classification for IgA nephropathy (IgAN), a disease with similar renal histology to HSN, appeared in 2009 [8]. The updated version of the Oxford classification (MEST-C) now considers five histologic parameters: mesangial hypercellullarity (M), endocapillary proliferation (E), segmental glomerulosclerosis (S), tubular atrophy and/or interstitial fibrosis (T), and crescents (C) [9]. However, in pediatric HSN, the feasibility of MEST-C requires still further research [7]. Furthermore, we recently introduced a semiquantitative classification (SQC) for histologic evaluation of HSN [10].

In this study, we evaluated ISKDC, SQC, and MEST-C scores in sequential renal biopsy specimens. Additionally, from diagnostic kidney biopsies, we analyzed immunohistochemically the expression of pro-fibrotic and inflammatory cell markers. Alpha-smooth muscle actin (α-SMA) and vimentin are pro-fibrotic markers, whereas P-selectin glycoprotein ligand-1 (PSGL-1) is a marker for inflammatory cells; early detection of the processes featuring these markers could prove important in enabling early treatment before the development of fibrosis. The aim of our study was to evaluate the prognostic value of (1) the ISKDC, SQC, and MEST-C classifications and (2) the expression of pro-fibrotic and inflammatory markers. Our hypothesis was that expression of these markers in the diagnostic kidney biopsies correlates with the findings in the follow-up renal biopsy, and that their expression is higher in HSN patients than in control samples from normal kidneys.

## Materials and methods

### Study design

The study comprises 26 childhood-onset (< 17 years) patients with HSN with an available follow-up renal biopsy specimen from Oulu University Hospital (1985–2005), Helsinki University Hospital (2000–2010) and a nationwide study [11]. A detailed description of the study population exists in our previous study, which analyzed the role of diagnostic renal biopsies for predicting outcomes in 53 HSN patients [10]. This study was approved by the ethics committee of Helsinki University Hospital (164/13/03/03/2016).

### Clinical data

Definition of proteinuria was urine protein to creatinine ratio (UP/C) > 20 g/mol, daily urine protein excretion (dU-Prot) > 200 mg, or a positive urine dipstick test (1+ to 3+). UP/C values at the time of the renal biopsies were converted into dU-Prot [12], and values over 40 mg/h/m^2^ denoted nephrotic-range proteinuria. Definition of hematuria was > 20 red blood cells/10E6/L or a positive urine dipstick test (1+ to 3+). Glomerular filtration rate was estimated (eGFR) with the bedside Schwartz equation [13]. Medical records provided data from the time of the diagnostic and follow-up biopsies and the last follow-up. Time from the onset of nephritis (proteinuria and/or hematuria) to renal biopsy and initiation of treatment was calculated. Patients (*n* = 2) who had not received immunosuppressive therapy were not included in the treatment delay analyses. Follow-up time was the period from HSP-diagnosis to the latest follow-up visit or to the start of renal replacement therapy. Indication for the diagnostic renal biopsy was either nephrotic-state proteinuria or persistence of proteinuria and/or hematuria up to 6–8 weeks. The 26 patients formed two groups at follow-up renal biopsy: patients without proteinuria (group I; *n* = 11) and with proteinuria (group II; *n* = 15). Eleven patients had no proteinuria at follow-up biopsy: nine of them underwent follow-up biopsy as part of a previous trial in accordance with the study protocol [11], one due to hematuria, and one for control purposes.

### Outcome

Outcome assessment at the last follow-up was as follows: outcome A (healthy)—no signs of renal disease; outcome B (minor urinary abnormalities)—UP/C = 20–100 g/mol and/or microscopic hematuria and/or ongoing ACE-I treatment; outcome C (active renal disease)—UP/C > 100 g/mol and/or ongoing immunosuppressive treatment; outcome D (reduced renal function)—eGFR < 60 mL/min/1.73 m^2^. Outcomes A + B were categorized as favorable outcome and outcomes C + D as unfavorable outcome.

### Renal biopsy classifications

Renal pathologists blinded to the patients’ medical history re-evaluated the biopsies with the ISKDC classification, SQC, and MEST-C. A detailed explanation of SQC parameters exists in our previous study [10]; the classification is also visible in online Table S1. Briefly, SQC comprises 14 renal histologic parameters and has a maximum score of 26 points; it divides into activity (maximum 9 points) and chronicity indices (maximum 16 points). In addition, a tubulointerstitial (including all active and chronic tubular and interstitial parameters) index can be calculated (maximum 5 points). The MEST-C scoring system of the Oxford classification includes five parameters and is defined as follows: M (mesangial hypercellularity defined as more than four mesangial cells in any mesangial area) as M0 (< 50% of glomeruli with mesangial hypercellularity) or M1 (> 50%); E (endocapillary proliferation) as E0 (absent) or E1 (present); S (segmental glomerulosclerosis) as S0 (absent) or S1 (present); T (tubular atrophy and/or interstitial fibrosis) as T0 (0–25% of cortical area affected), T1 (26–50%), or T2 (> 50%) and C (crescents) as C0 (absent), C1 (at least 1 crescent, but crescents in a maximum of 25% of glomeruli) or C2 (> 25%). In addition, total MEST-C score was calculated (sum of all five MEST-C parameters).

### Immunohistochemistry and microscopy

Diagnostic renal biopsy specimens, formalin-fixed and paraffin-embedded, were cut into 4–5-μm-thick slices. They underwent a conventional immunohistochemical staining process. Primary antibodies were used against α-SMA (clone 1A4, diluted 1:400, Dako Denmark A/S, Glostrup, Denmark), vimentin (clone 3B4, 1:200, Dako), and PSGL-1 (sc-13535, 1:500, Santa Cruz Biotechnology, Inc., Dallas, TX, USA). Eighteen (69%) biopsies were successfully stained with α-SMA, 19 (73%) with vimentin, and 17 (65%) with PSGL-1. Negative controls containing no primary antibodies were incubated in phosphate-buffered saline. Normal kidneys, originally removed with an intent to use as kidney transplants, served as control specimens. Supplementary material contains images (Figures S1–S3) of typical expression of the analyzed molecules in HSN patients and in control specimens.

The microscopy tool used was Zeiss AX10. Analyses of the HSN biopsy specimens involved all glomeruli (with × 20 magnification) and as many microscopic fields as possible from the cortical tubulointerstitium (× 40). Analysis of each control specimen included 30 randomly selected glomeruli (× 20) and 30 randomly selected, non-overlapping tubulointerstitial microscopic fields (× 40). Zeiss Zen software (Carl Zeiss AG, Oberkochen, Germany) performed calculation of the tubulointerstial area of positive α-SMA and vimentin staining. The number of PSGL-1 staining patches was counted from the tubulointerstitium and glomeruli. In evaluation of the staining results, scores of all microscopic fields and glomeruli were totaled separately for control specimens, group I, and group II. To assess correlation with staining results and biopsy findings in HSN patients, mean of the microscopic fields’ affected areas (α-SMA, vimentin) and the mean number of staining patches in the tubulointerstitium and glomeruli (PSGL-1) represented the scores for each patient. Patients’ clinical data was not available when conducting the immunohistochemical analyses.

### Statistical analyses

The statistical analysis software used was IBM SPSS version 22 (IBM Corp, Armonk, NY). Continuous variables are reported as means and standard deviations (SD) (normal distribution) or medians and interquartile ranges (IQR) (non-normal distribution), and analyzed with Student’s *t* test (normal distribution) or Mann–Whitney *U* test (non-normal distribution). Categorical variables are reported as numbers and percentages, and analyzed with Fisher’s exact test. Change in histologic variables from the diagnostic biopsy to the follow-up biopsy was analyzed with McNemar test (MEST-C parameters) or Wilcoxon-signed rank test (SQC parameters). Spearman rank correlations were calculated between staining results, biopsy findings, and biopsy delays. Results from linear regression are reported as regression coefficients (*B* value) with 95% confidence intervals. Predictors with *p* < 0.2 in univariable analysis were selected to the multivariable analysis. The level of statistical significance was *p* < 0.05.

## Results

### Patient characteristics and outcome

Table 1 provides baseline patient characteristics. Patients in group II were older than those in group 1; no other statistically significant differences existed between the groups at the time of the diagnostic renal biopsy. Median time from onset of nephritis to the diagnostic renal biopsy was 59 (IQR 36–71) days in patients without proteinuria (group I) and 33 (IQR 19–60) days in proteinuric patients (group II) (*p* = 0.17). Median time from renal biopsy to immunosuppressive treatment was 7 (IQR 4–8) days and 7 (IQR 2–16) days in groups I and II, respectively (*p* = 0.94).

**Table 1:** Baseline characteristics at the diagnostic renal biopsy, ISKDC findings of the diagnostic biopsy, and used therapies

	All (*n* = 26)	Group I (*n* = 11)	Group II (*n* = 15)	*p* value
Male, *n* (%)	17 (65%)	5 (45%)	12 (80%)	0.10
Age (years)	9.8 ± 3.4	7.7 ± 2.6	11.2 ± 3.1	0.006
Plasma creatinine (μmol/L)	52 (45–58)	51 (44–55)	53 (46–62)	0.30
eGFR (mL/min/1.73 m^2^)	99 (87–109)	90 (87–108)	100 (89–108)	0.80
Plasma albumin (g/L)	29.3 ± 8.8	30.4 ± 8.2	28.7 ± 9.4	0.66
dU-Prot^a^ (g/day)	4.8 (1.6–7.7)	3.8 (1.0–5.8)	4.8 (1.8–10.2)	0.50
dU-Prot^a^ (mg/h/m^2^)	140 (69–328)	136 (52–233)	150 (77–332)	0.72
Nephrotic-range proteinuria (> 40 mg/h/m^2^), *n* (%)	20 (77%)	8 (73%)	12 (80%)	> 0.99
Hematuria, *n* (%)	26 (100%)	11 (100%)	15 (100%)	> 0.99
ISKDC				
I II IIIa IIIb IV V	0 6 5 12 2 1	0 4 1 6 0 0	0 2 4 6 2 1	
SQC activity score	4 (2–7)	4 (1–4)	6 (4–7.5)	0.018
SQC chronicity score	3 (2–4)	3 (1.5–3.5)	3 (2.5–4)	0.37
SQC tubulointerstitial score	0 (0–1)	0 (0–0.5)	0 (0–1)	0.31
IT, *n* (%)	24 (92%)	9^b^ (82%)	15^c^ (100%)	0.17
ACE-I, *n* (%)	21 (81%)	8 (73%)	13 (87%)	0.62

Group I represents patients without proteinuria and group II patients with proteinuria at the follow-up biopsy*. eGFR*, estimated glomerular filtration rate; *dU-Prot*, daily urine protein excretion; *IT*, immunosuppressive therapy; *ACE-I*, angiotensin-converting enzyme inhibitor

^a^For one patient in group I proteinuria at the diagnostic renal biopsy was measured only as a negative dipstick test and was not included in the analyses

^b^Initial immunosuppressive therapies in group I were methylprednisolone pulses (*n* = 6) and cyclosporine A (*n* = 3). Two patients had not received immunosuppressive therapy

^c^Initial immunosuppressive therapies in group II were methylprednisolone pulses (*n* = 8), cyclosporine A (*n* = 4), azathioprine (*n* = 1), cyclophosphamide (*n* = 1), and oral prednisone (*n* = 1)

Table 2 presents patient characteristics at the follow-up biopsy and at last follow-up. Follow-up biopsy was performed a median of 2.2 (IQR 2.0–2.3) years after the diagnostic biopsy in group I and 2.1 (1.7–2.2) years in group II (*p* = 0.31). At the follow-up biopsy, plasma creatinine levels were significantly higher in group II than in group I, but there was no statistically significant difference in eGFR. Clinical and biopsy characteristics according to the outcome (favorable/unfavorable) are presented in the Supplementary material (Table S2). At the follow-up biopsy, patients with unfavorable outcome had significantly higher proteinuria than those with favorable outcome (median dU-Prot 4.7 vs. 0.2 g/day; *p* < 0.001).

**Table 2:** Patient characteristics at the follow-up renal biopsy, ISKDC findings of the follow-up biopsy, and outcome at the last follow-up visit

	All (*n* = 26)	Group I (*n* = 11)	Group II (*n* = 15)	*p* value
At the follow-up biopsy				
Age (years)	12.6 ± 4.6	11.1 ± 5.4	13.8 ± 3.7	0.14
Plasma creatinine (μmol/L)	58 (41–67)	53 (38–58)	66 (53–74)	0.024
eGFR (mL/min/1.73 m^2^)	98 (89–121)	103 (96–126)	94 (82–108)	0.069
dU-Prot^a^ (g/day)	0.3 (0.1–1.2)	0.10 (0.91–0.11)	0.9 (0.3–3.3)	NA^b^
Hematuria, *n* (%)	16 (62%)	5 (45%)	11 (73%)	0.23
ISKDC				
I II IIIa IIIb IV V	1 16 3 4 1 1	1 9 0 1 0 0	0 7 3 3 1 1	
SQC activity score	2 (1–2)	1 (1–2)	2 (1–4,5)	0.033
SQC chronicity score	7 (5–9)	6 (3.5–7)	9 (7–10.5)	0.013
SQC tubulointerstitial score	3 (2–3)	3 (1–3)	3 (2–4)	0.096
At the last follow-up				
Age (years)	19.1 ± 6.7	16.0 ± 6.5	21.4 ± 5.9	0.035
Follow-up (years)	8.6 (6.4–11.3)	6.8 (6.2–8.2)	9.0 (8.3–12.8)	0.041
Plasma creatinine (μmol/L)	75 (58–87)	58 (51–71)	77 (75–123)	0.003
eGFR^c^ (mL/min/1.73 m^2^)	86 (73–96)	96 (86–121)	83 (38–86)	0.011
Outcome				
A B C D	15 6 1 4	9 2 0 0	6 4 1 4	

Group I represents patients without proteinuria and group II patients with proteinuria at the follow-up biopsy. *eGFR*, estimated glomerular filtration rate; *dU-Prot*, daily urine protein excretion; *NA*, not applicable

^a^*n* = 21 (8 in group I and 13 in group II; for 3 patients in group I and 2 patients in group II proteinuria was measured only as a dipstick test or as U-Prot; these patients were not included in the analysis)

^b^Group I comprises patients with no proteinuria at the follow-up renal biopsy

^c^*n* = 25 (11 in group I and 14 in group II)

At the last follow-up, patients without proteinuria (group I) had significantly lower creatinine and higher eGFR than proteinuric patients (group II). Fifteen (58%) patients belonged to outcome group A, 6 (23%) to outcome group B, 1 (4%) to outcome group C, and 4 (15%) to outcome group D. Five patients had unfavorable outcome: one had ongoing cyclosporine A treatment due to persistent proteinuria, one had reduced renal function (eGFR 37 mL/min/1.73 m^2^), two developed end-stage kidney disease, and one had succumbed due to HSN [14]. None in group I developed unfavorable outcome compared to five patients (33%) in group II (*p* = 0.053).

### SQC and ISKDC

**Fig. 1:**
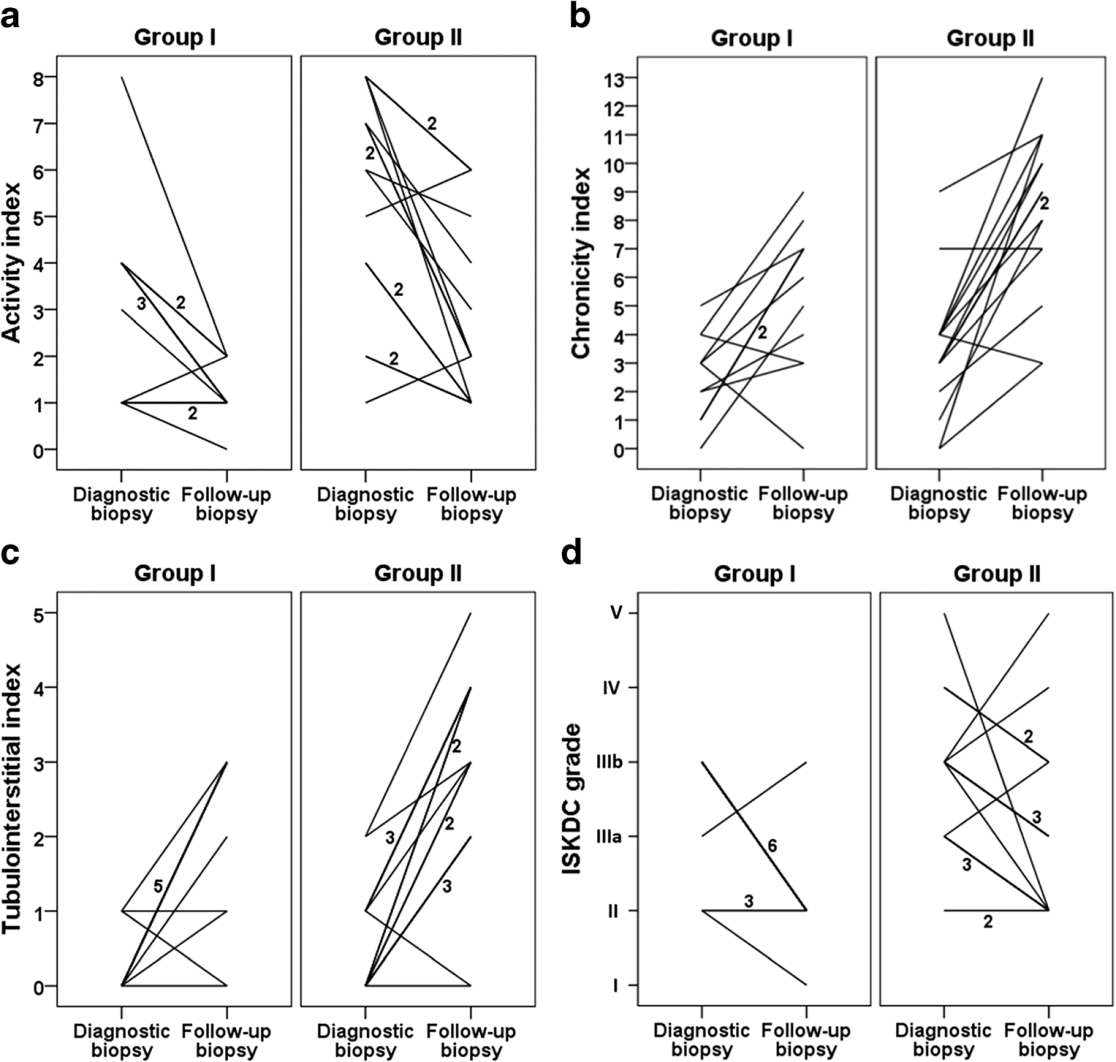
Changes in the activity (**a**), chronicity (**b**), and tubulointerstitial scores (**c**) of the semiquantitative classification (SQC), and in the International Study of Kidney Diseases in Children classification (ISKDC) grades (**d**) between diagnostic and follow-up biopsy presented individually in 26 Henoch–Schönlein nephritis patients. The number of patients with decreased activity score (*p* = 0.62), decreased ISKDC grade (*p* > 0.99), increased chronicity score (*p* > 0.99), or increased tubulointerstitial score (*p* = 0.62) did not differ between groups I and II. Numbers in figures denote the number of patients with similar change in respective SQC score or ISKDC grade

In the diagnostic and follow-up biopsies of all 26 patients, median activity scores were 4 (IQR 2–7) and 2 (IQR 1–2) (*p* < 0.001), median chronicity scores 3 (IQR 2–4) and 7 (IQR 5–9) (*p* < 0.001), and median tubulointerstitial scores 0 (IQR 0–1) and 3 (IQR 2–3) (*p* < 0.001), respectively. Activity scores in the diagnostic and follow-up biopsy and chronicity score in the follow-up biopsy were significantly higher in group II than in group I (Tables 1 and 2). Figure 1 illustrates individually in each patient the changes in the SQC activity, chronicity, and tubulointerstitial scores, and in the ISKDC grades between diagnostic and follow-up biopsy. Between the biopsies, SQC activity score decreased in 21 (81%) patients and ISKDC grades in 17 (65%), whereas chronicity score increased in 22 (85%) patients and tubulointerstitial score in 21 (81%). Changes occurred similarly in groups I and II (Fig. 1). In all five patients with unfavorable outcome, activity scores decreased whereas chronicity and tubulointerstitial scores increased (Table S2). SQC activity and chronicity scores in the follow-up biopsy showed no significant correlation with time from disease onset to treatment and did not differ significantly according to the initial treatment (data not shown). In multiple regression, activity score in the diagnostic biopsy was an independent predictor of the chronicity score in the follow-up biopsy (Table 3). Median number of glomeruli per biopsy was 21 (IQR 12–25) in the diagnostic biopsy and 15 (IQR 12–22) in the follow-up biopsy.

**Table 3:** Linear regression results for chronicity score in the follow-up biopsy

	Univariable			Multivariable		
	*B*	95% CI	*p* value	*B*	95% CI	*p* value
Gender (male vs. female)	− 0.39	− 3.12 to 2.34	0.77			
Age at onset (years)	0.35	− 0.19 to 0.71	0.062	− 0.005	− 0.30 to 0.29	0.97
dU-Prot^a^ (mg/h/m^2^)	− 0.004	− 0.014 to 0.005	0.33			
eGFR^a^ (mL/min/1.73 m^2^)	− 0.011	− 0.064 to 0.041	0.66			
SQC activity score^a^	0.94	0.60 to 1.28	< 0.001	0.74	0.34 to 1.12	0.001
SQC chronicity score^a^	0.43	− 0.19 to 1.05	0.17	0.42	− 0.032 to 0.88	0.067
M1^a^	0	− 2.68 to 2.68	> 0.99			
E1^a^	− 1.35	− 4.68 to 1.98	0.41			
S1^a^	0.11	− 2.50 to 2.72	0.93			
C1–2^a^	2.38	− 0.008 to 4.76	0.051	0.86	− 1.23 to 2.95	0.40

T1 was not present in the diagnostic biopsies and thus was not included in the analyses. Since crescents are included also in the SQC activity score, we performed multivariable analysis with age at onset, SQC activity score, and SQC chronicity score (i.e., excluding C1–2 from the analysis). Results did not markedly change (data not shown). *B*, regression coefficient; *CI*, confidence interval; *dU-Prot*, daily urine protein excretion; *eGFR,* estimated glomerular filtration rate; *M*, mesangial hypercellularity; *E*, endocapillary proliferation; *S*, segmental glomeruloscrelosis; *T*, tubular atrophy and/or interstitial fibrosis; *C*, crescents

^a^At the diagnostic biopsy

### MEST-C

Table 4 shows MEST-C scores in the diagnostic and follow-up biopsies. Between the biopsies, S1 (from 38 to 79%; *p* = 0.006) and T1–2 (from 0 to 13%; *p* = 0.25) scores increased, whereas E1 (from 83 to 13%; *p* < 0.001) and C1–2 (from 63 to 25%; *p* = 0.022) scores decreased. M1 was similarly present in both biopsies (33%; *p* > 0.99). Table 4 also reports biopsies where tubular atrophy and/or interstitial fibrosis (*T* score) was present, but for less than 25% of the cortical area (marked as *T* > 0% in Table 3). *T* > 0% score increased from 4 to 67% between the diagnostic and follow-up biopsy (*p* < 0.001). Marked differences between group I and group II occurred especially in the follow-up biopsies’ presence of C1–2 (0% vs. 40%, *p* = 0.052) and E1 (0% vs. 20%, *p* = 0.27). Baseline characteristics for patients with and without C1–2 at the follow-up biopsy were: age 12.1 years (IQR 7.6–14.1) vs. 9.0 years (IQR 7.6–11.8) (*p* = 0.31), proteinuria 284 mg/m^2^/h (IQR 106–336) vs. 131 mg/m^2^/h (52–255) (*p* = 0.35), and eGFR 94 mL/min/1.73 m^2^ (IQR 85–101) vs. 102 mL/min/1.73 m^2^ (IQR 89–110) (*p* = 0.42), respectively. Initial treatment of patients with C1–2 at the follow-up biopsy was methylprednisolone pulses (*n* = 4), cyclosporine A (*n* = 1), and cyclophosphamide (*n* = 1).

**Table 4:** MEST-C scoring according to the Oxford classification

	Diagnostic biopsy				Follow-up biopsy			
	All	Group I	Group II	*p* value	All	Group I	Group II	*p* value
M1, *n* (%)	8 (33%)	2 (22%)	6 (40%)	0.66	8 (33%)	2 (22%)	6 (40%)	0.66
E1, *n* (%)	20 (83%)	7 (78%)	13 (87%)	0.62	3 (13%)	0	3 (20%)	0.27
S1, *n* (%)	9 (38%)	4 (44%)	5 (33%)	0.68	19 (79%)	6 (67%)	13 (87%)	0.33
T1–2, *n* (%)	0	0	0	NA	3 (13%)	1 (11%)	2 (13%)	> 0.99
T > 0%^a^, *n* (%)	1 (4%)	0	1 (7%)	> 0.99	16 (67%)	5 (56%)	11 (73%)	0.41
C1, *n* (%)	9 (38%)	5 (56%)	4 (27%)	0.21	5 (21%)	0	5 (33%)	0.12
C2, *n* (%)	6 (25%)	0	6 (40%)	0.052	1 (4%)	0	1 (7%)	> 0.99
C1–2, *n* (%)	15 (63%)	5 (56%)	10 (67%)	0.68	6 (25%)	0	6 (40%)	0.052
Total MEST-C score	2.5 (2–3)	2 (2–3)	3 (2–3)	0.39	1 (1–2.5)	1 (1–1)	2 (1–3.5)	0.10

48/52 renal biopsy specimens (combined diagnostic and follow-up renal biopsy) were available for MEST-C analysis. Group I represents patients without proteinuria and group II patients with proteinuria at the follow-up biopsy. *M*, mesangial hypercellularity; *E*, endocapillary proliferation; *S*, segmental glomeruloscrelosis; *T*, tubular atrophy and/or interstitial fibrosis; *C*, crescents; *NA*, not applicable

^a^Comprises patients whose renal biopsies showed tubular atrophy and/or interstitial fibrosis affecting > 0% of the renal cortical area

### Immunohistochemistry

**Fig. 2:**
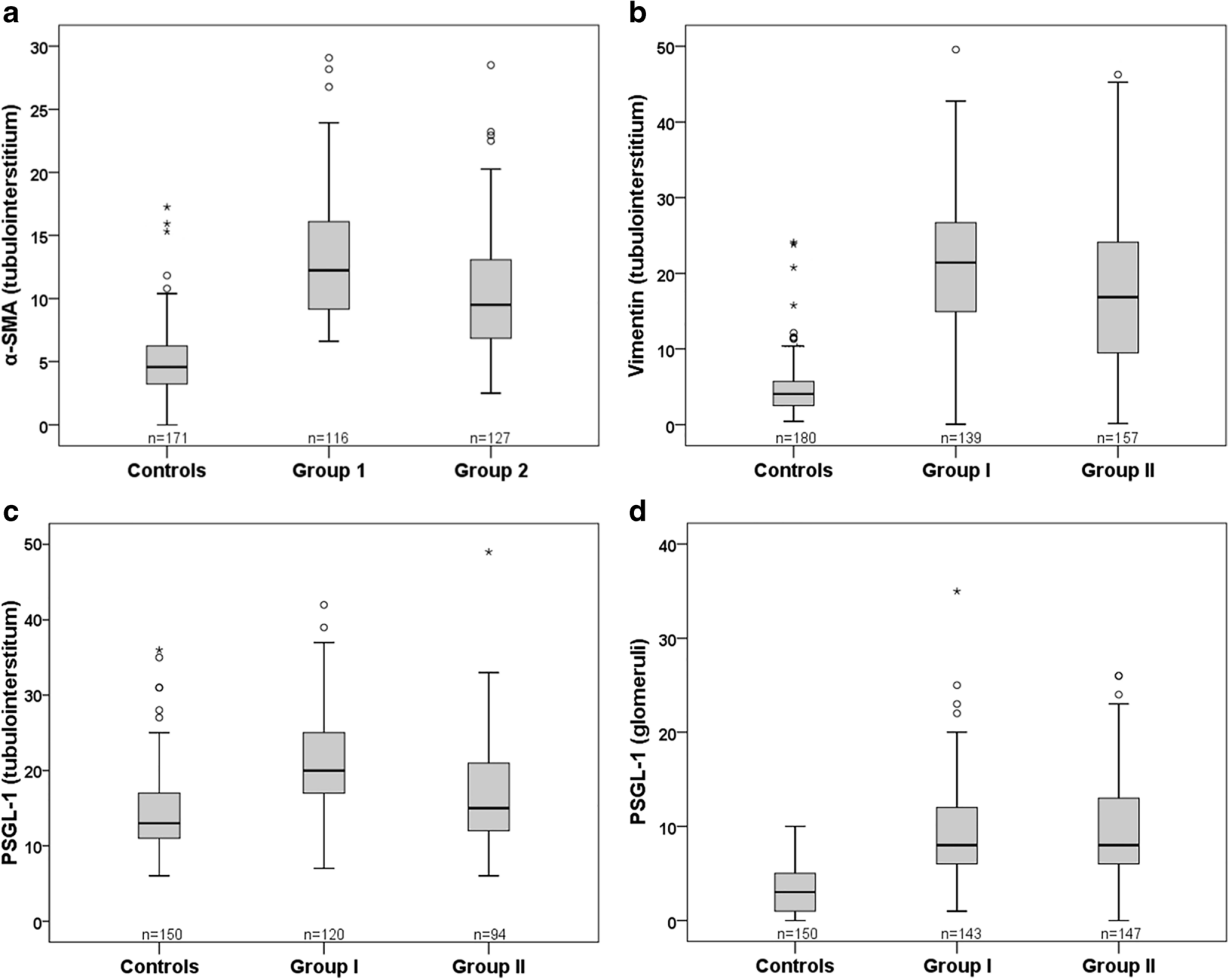
Scores for tubulointerstitial alpha-smooth muscle actin (α-SMA) (**a**), vimentin (**b**), and P-selectin glycoprotein ligand-1 (PSGL-1) (**c**), as well as for glomerular PSGL-1 (**d**), in normal kidney specimens and in Henoch–Schönlein nephritis patients without proteinuria (group I) and with proteinuria (group II) at the follow-up renal biopsy. Numbers in figures denote the total number of microscopic fields or glomeruli in each group

Expression of α-SMA, vimentin, and PSGL-1 was higher in HSN biopsies than in control specimens (combined HSN groups I and II vs. controls, *p* < 0.001 for all markers). Tubulointerstitial scores (α-SMA, vimentin, PSGL-1) were higher in group I than in group II (*p* < 0.001 for all markers) (Fig. 2). In glomeruli, PSGL-1 expression in groups I and II was similar (*p* = 0.85). Table 5 contains correlations between staining results and SQC activity, chronicity, and tubulointerstitial indices. α-SMA showed positive correlations with all parameters and significant correlation with follow-up biopsies’ tubulointerstitial index. Vimentin showed significant negative correlation with chronicity and tubulointerstitial index of the follow-up biopsy. PSGL-1 exhibited no significant correlation with the SQC indices. No staining showed significant correlation with the time from the onset of nephritis to the diagnostic renal biopsy (α-SMA: *r* = 0.22, *p* = 0.38; vimentin: *r* = -0.066, *p* = 0.79; PSGL-1 in glomeruli: *r* = -0.13, *p* = 0.62; PSGL-1 in tubulointerstitium: *r* = 0.065, *p* = 0.83).

**Table 5:** Correlation with mean expression of α-SMA, vimentin, and PSGL-1 scores in the diagnostic biopsy and activity, chronicity, and tubulointerstitial indices of the diagnostic and follow-up renal biopsy

	Diagnostic biopsy			Follow-up biopsy		
	AI	CI	TI	AI	CI	TI
α-SMA in tubulointerstitium	*r* = 0.26 *p* = 0.29	*r* = 0.27 *p* = 0.29	*r* = 0.37 *p* = 0.13	*r* = 0.35 *p* = 0.15	*r* = 0.27 *p* = 0.29	*r* = 0.55 *p* = 0.019
Vimentin in tubulointerstitium	*r* = − 0.34 *p* = 0 .15	*r* = − 0.37 *p* = 0.12	*r* = – 0.27 *p* = 0.27	*r* = − 0.27 *p* = 0.26	*r* = − 0.60 *p* = 0.007	*r* = − 0.53 *p* = 0.019
PSGL-1 in tubulointerstitium	*r* = 0.081 *p* = 0.78	*r* = 0.067 *p* = 0.82	*r* = 0.13 *p* = 0.65	*r* = – 0.012 *p* = 0.97	*r* = − 0.091 *p* = 0.76	*r* = − 0.044 *p* = 0.88
PSGL-1 in glomeruli	*r* = − 0.10 *p* = 0.70	*r* = 0.26 *p* = 0.32	*r* = 0.06 *p* = 0.82	*r* = − 0.02 *p* = 0.94	*r* = − 0.16 *p* = 0.53	*r* = − 0.36 *p* = 0.15

*AI*, activity index; *CI*, chronicity index; *TI*, tubulointerstitial index; *α-SMA*, alpha-smooth muscle actin; *PSGL-1*, P-selectin glycoprotein ligand-1

## Discussion

The aim of our study was to evaluate the prognostic value of three histologic classifications: ISKDC, SQC, and MEST-C. We also evaluated whether pro-fibrotic and inflammatory markers in diagnostic renal biopsy specimens predicted clinical outcomes and lesions in follow-up renal biopsies. Between the diagnostic and follow-up biopsy, active changes decreased and chronic changes increased regardless of the clinical symptoms. Nonetheless, patients with proteinuria at the follow-up biopsy (group II) had higher SQC activity scores in the diagnostic biopsy and higher activity and chronicity scores at the follow-up biopsy than patients without proteinuria (group I). The studied pro-fibrotic and inflammatory markers were higher in HSN biopsy specimens than in controls, but showed no value as predictors of prolonged proteinuria.

We divided the patients according to proteinuria at the follow-up biopsy, since prolonged proteinuria is associated with worsened prognosis [15, 16]. In accordance with these reports, all five patients with poor outcome in our study had prolonged proteinuria. In most patients, regardless of the proteinuria, activity scores decreased and chronicity scores increased from diagnostic biopsy to follow-up biopsy. This was also evident in MEST-C scores: between the biopsies E1 decreased, whereas S1, T1–2, and T > 0% increased. Regarding these changes in activity and chronicity scores in serial HSN biopsies, previous studies have shown comparable results [17–19]. Consistent with the findings of Shin et al. [17], the follow-up biopsies of 21 patients with favorable outcome contained chronic histologic changes. This suggests that even with favorable therapeutic outcome, HSN patients require long-term follow-up since the increased histological chronicity may impact the long-term prognosis. Furthermore, since most of our patients were treated with immunosuppressive drugs and angiotensin-converting enzyme inhibitors, progression of the chronicity signs can be considered as an insufficient response to the treatment.

Based on our results, MEST-C parameters in pediatric HSN seem feasible. MEST-C originates from adult IgAN patient data and thus some modifications to the classification may prove necessary for the evaluation of pediatric HSN. For example, tubulointerstitial lesions of MEST-C (i.e., T1 and 2) in our cohort were rare. They were, however, more common after inclusion of biopsies with less than 25% of affected area. Progression of tubulointerstitial lesions associates with a longer duration of proteinuria [20], and it could therefore prove important to consider all lesions affecting the tubulointerstitium and not only those exceeding 25% of the renal cortex area.

C1–2 were present in both groups’ diagnostic biopsies, but were only seen in the follow-up biopsies in group II. The persistence of crescents thus seems to associate with prolonged proteinuria, supporting results from other studies that treatment should be commenced before fibrotization of crescents [19]. Active treatment is also supported by the finding that active lesions in the diagnostic biopsy predicted chronic lesions in the follow-up biopsy. M1 was similarly present in both biopsies. Similar results were observed in adult IgAN patients after a 6-month treatment with mycophenolate mofetil and prednisone [21]. M1 thus seem not to be a flexible sign of clinical improvement and may suggest that the underlying pathogenetic event is still present. In HSN, the clinical meaning (and whether they need further therapy) of the persistent M1 and increased S1/T1-2 findings is unknown. It is possible that the renal damage continues despite minimal clinical signs and that the eGFR has remained stable due to the insufficient follow-up.

Oxford classification has been validated to predict outcomes in IgAN, and in a recent study, M1, S1, and T1–2 were predictive of progression decades after the renal biopsy [22]. Previous studies on the feasibility of MEST-C in pediatric HSN have suggested that the chronic lesions (S1 and T1–2) correlate with poor outcome. Xu et al. reported that segmental glomerulosclerosis (S1) was associated with a poor renal outcome in univariate analysis, whereas no significant association existed with other parameters. T1 and 2 lesions were, however, negatively associated with the risk of proteinuria remission while other lesions showed no significant association. Interestingly, the number of biopsies with especially E1 and T1 and 2 in the study by Xu et al. [23] differs from ours. This may reflect inter-observer variation between pathologists or differences in patient material and biopsy timing. T1–2 and S1 associated with worsened renal survival also in a recent study of 75 biopsied pediatric HSN patients [24]. In two studies with adult HSN patients, E1 also predicted outcome, with or without S1 [25, 26]. In our study, marked differences between groups I and II occurred in C1–2 and E1 in the follow-up biopsy, although no MEST-C parameter differed significantly. Significant differences existed, however, in the SQC subclasses as activity indices in both biopsies and chronicity index in the follow-up biopsy were higher in proteinuric patients (group II) than in non-proteinuric patients (group I). This may indicate that the evaluation of several histologic variables together could be more advantageous than the assessment of single separate variables.

Vimentin and α-SMA are markers of fibroblast activation [27], whereas PSGL-1 is an inflammatory cell marker [28]. Vimentin and α-SMA associate with disease progression in IgAN [29], but reports on the expression of these molecules in HSN are scarce. Kawasaki et al. studied the expression of α-SMA and c-MET in HSN. Hepatocyte growth factor, which has anti-fibrogenetic effects, uses c-MET as a receptor. In the study by Kawasaki et al., the expression of α-SMA in the first renal biopsy correlated with the chronicity index of the second biopsy in patients with crescents (ISKDC ≥ III) in the first renal biopsy. c-MET showed no significant correlation with activity or chronicity indices [18]. Our results are consistent with those of Kawasaki et al. concerning α-SMA since in our study, tubulointerstitial α-SMA had significant positive correlation with the tubulointerstitial index—which reflects mainly chronic changes—of the follow-up biopsy. Nonetheless, α-SMA showed no significant correlation with the chronicity index itself. Tubulointerstitial scores for α-SMA, vimentin, and PSGL-1 were surprisingly higher in patients without proteinuria at the follow-up biopsy, but all three markers showed great overlap between the HSN groups (Fig. 2). We were not able to analyze the expression of α-SMA and vimentin from glomeruli. This was due to heavy staining, which precluded any quantitative or semiquantitative analysis.

Our study has limitations. First, most patients had received therapy, which had most likely altered the disease course and possibly hampered the prognostic significance of renal biopsies overall [30]. Second, the low number of patients with unfavorable outcome limits the interpretation of results from comparison between patients with favorable and unfavorable outcome. These two limitations are probably due to the active treatment of severe HSN in Finland [31]. Third, histologic evaluation is always subject to inter-rater variability. The inter-rater variability of SQC, assessed in our previous study, showed fair to good reproducibility in a randomly chosen subset of ten biopsies [10].

Our study has strengths. First, we were able to analyze follow-up biopsies from patients with and without proteinuria at the follow-up biopsy. This allowed us to evaluate the biopsies in relation to the patients’ clinical status. Second, data on the feasibility of the MEST-C in pediatric HSN is scarce, and in our study, the follow-up biopsies also underwent evaluation with MEST-C classification. Third, the median follow-up time in our study was 8.6 years.

Our results suggest that from diagnostic to follow-up renal biopsy, active signs decrease and chronic signs increase regardless of the clinical status, and thus follow-up biopsies provide limited additive information to the ongoing clinical symptoms (especially severe proteinuria) in HSN outcome prediction. Nonetheless, all severe HSN patients, even after a good therapeutic result, require long-term follow-up. Compared to control specimens, HSN biopsies showed over-expression of the pro-fibrotic and inflammatory markers, but none were associated with prolonged proteinuria.

## Electronic supplementary material

ESM 1 (PDF 271 kb)

ESM 2 (PDF 591 kb)

ESM 3 (PDF 607 kb)

ESM 4 (PDF 591 kb)

ESM 5 (PDF 415 kb)
