# Supplementary material for: Prediction of renal outcome in Henoch–Schönlein nephritis based on biopsy findings
Source: Pediatr Nephrol. 2019 Dec 3;35(4):659–68. doi: 10.1007/s00467-019-04415-3 (PMC7056733; doi:10.1007/s00467-019-04415-3)
Supplement: Supplementary file 1 — (PDF 271 kb) [file 467_2019_4415_MOESM1_ESM.pdf]

Article title: Prediction of renal outcome in Henoch-Schönlein nephritis based on biopsy findings

Journal: Pediatric Nephrology

Author names: Mikael Koskela, Elisa Ylinen, Helena Autio-Harmainen, Heikki Tokola, Päivi Heikkilä, Jouko Lohi, Hannu Jalanko, Matti Nuutinen, Timo Jahnukainen

Corresponding author: Mikael Koskela; Children's Hospital, Pediatric Research Center, University of Helsinki, Helsinki University Hospital, Helsinki, Finland; e-mail address: mikael.koskela@helsinki.fi

Table S1. Histologic parameters of the semiquantitative classification (SQC)

	Active or chronic	Scoring
**Glomerular parameters**		
Lobulation	active	0-1[a]
Mesangial proliferation	active	0-1[a]
Crescents		
cellular	active	0-3[b]
fibrous	chronic	0-3[b]
Adhesions	chronic	0-3[b]
Fibrinous thrombosis	active	0-3[b]
Global sclerosis	chronic	0-3[b]
Segmental sclerosis	chronic	0-2[c]
**Tubular parameters**		
Thickening of the basement membrane	chronic	0-1[a]
Complete atrophy	chronic	0-1[a]
Tubular dilatation	active	0-1[a]
**Interstitial parameters**		
Fibrosis	chronic	0-1[a]
Inflammation OR periglomerular inflammation	chronic	0-1[a]
**Capillary parameters**		
Arteriosclerosis OR arterial inflammation	chronic	0-1[a]
**Focal or diffuse mesangial proliferation**		0 for focal, 1 for diffuse

[a]0 = absent; 1 = present
[b]0 = 0% of glomeruli affected; 1 = 0-5% of glomeruli affected; 2 = 5-10% of glomeruli affected; 3 = >10% of glomeruli affected
[c]0 = 0% of glomeruli affected; 1 = 0-5% of glomeruli affected; 3 = >5% of glomeruli affected

SQC was originally published in: The ISKDC classification and a new semiquantitative classification for predicting outcomes of Henoch-Schonlein purpura nephritis. *Pediatr Nephrol* 2017; 32: 1201-1209.
