# Supplementary material for: Prediction of renal outcome in Henoch–Schönlein nephritis based on biopsy findings
Source: Pediatr Nephrol. 2019 Dec 3;35(4):659–68. doi: 10.1007/s00467-019-04415-3 (PMC7056733; doi:10.1007/s00467-019-04415-3)
Supplement: Supplementary file 3 — (PDF 607 kb) [file 467_2019_4415_MOESM3_ESM.pdf]

Figure S2

Article title: Prediction of renal outcome in Henoch-Schönlein nephritis based on biopsy findings
Journal: Pediatric Nephrology
Author names: Mikael Koskela, Elisa Ylinen, Helena Autio-Harmainen, Heikki Tokola, Päivi Heikkilä, Jouko Lohi, Hannu Jalanko, Matti Nuutinen, Timo Jahnukainen
Corresponding author: Mikael Koskela; Children's Hospital, Pediatric Research Center, University of Helsinki, Helsinki University Hospital, Helsinki, Finland; e-mail address: mikael.koskela@helsinki.fi

A

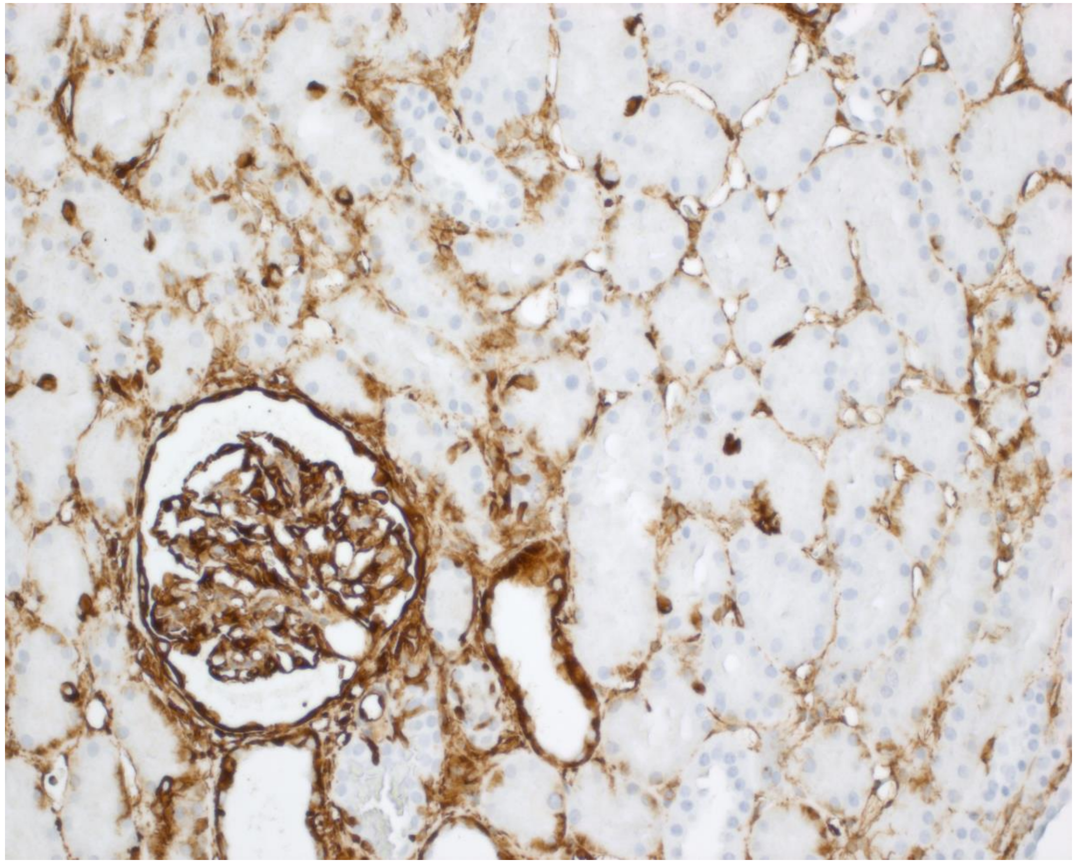

B

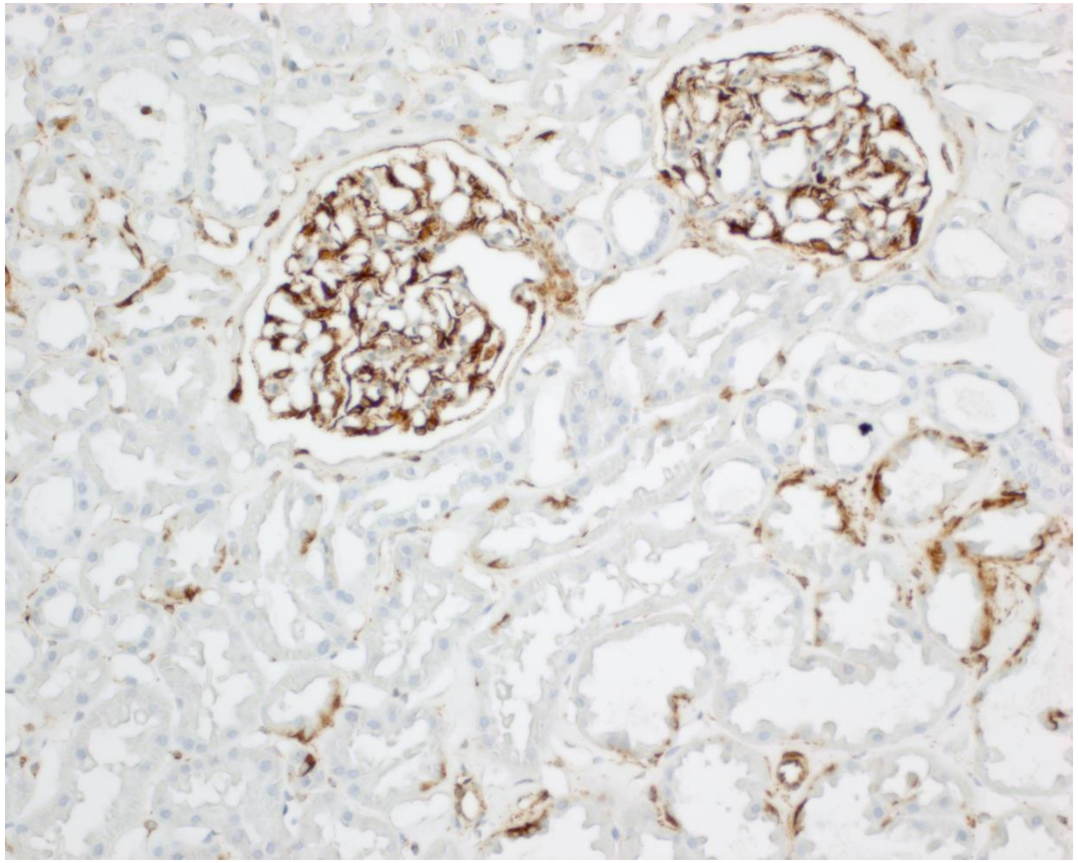

Expression of vimentin in Henoch-Schönlein nephritis (A) and in control specimen (B)
