# Supplementary material for: Prediction of renal outcome in Henoch–Schönlein nephritis based on biopsy findings
Source: Pediatr Nephrol. 2019 Dec 3;35(4):659–68. doi: 10.1007/s00467-019-04415-3 (PMC7056733; doi:10.1007/s00467-019-04415-3)
Supplement: Supplementary file 5 — (PDF 415 kb) [file 467_2019_4415_MOESM5_ESM.pdf]

Article title: Prediction of renal outcome in Henoch-Schönlein nephritis based on biopsy findings

Journal: Pediatric Nephrology

Author names: Mikael Koskela, Elisa Ylinen, Helena Autio-Harmainen, Heikki Tokola, Päivi Heikkilä, Jouko Lohi, Hannu Jalanko, Matti Nuutinen, Timo Jahnukainen

Corresponding author: Mikael Koskela; Children's Hospital, Pediatric Research Center, University of Helsinki, Helsinki University Hospital, Helsinki, Finland; e-mail address: mikael.koskela@helsinki.fi

Table S2 Clinical characteristics and biopsy findings in patients with favorable and unfavorable outcome

	Favorable outcome (n=21)	Unfavorable outcome (n=5)	p-value
**At the diagnostic biopsy**			
Age (years)	9.3 ± 3.4	11.7 ± 2.5	0.14
Plasma creatinine (μmol/L)	52 (45 – 56)	56 (48 – 88)	0.31
eGFR (mL/min/1.73m^2)	98 (88 – 109)	99 (65 – 101)	0.57
dU-Prot[a] (g/day)	2.5 (1.1 – 5.4)	10.4 (9.9 – 12.4)	0.004
Nephrotic-range proteinuria (>40 mg/h/m^2), n (%)	15 (71%)	5 (100%)	0.30
ISKDC			
I	0	0	
II	6	0	
IIIa	5	0	
IIIb	9	3	
IV	1	1	
V	0	1	
SQC activity score	4 (2 – 5)	8 (7 – 8)	0.003
SQC chronicity score	3 (1 – 4)	4 (4 – 4)	0.096
SQC tubulointerstitial score	0 (0 – 1)	1 (1 – 2)	0.028
MEST-C[b]			
M1, n (%)	5 (26%)	3 (60%)	0.29
E1, n (%)	15 (79%)	5 (100%)	0.54
S1, n (%)	7 (37%)	2 (40%)	>0.99
T1-2, n (%)	0	0	NA
T>0%, n (%)	1 (5%)	0	>0.99
C1, n (%)	9 (47%)	0	0.12
C2, n (%)	2 (11%)	4 (80%)	0.006
C1-2, n (%)	11 (58%)	4 (80%)	0.62
Total MEST-C score	2 (2 – 3)	4 (3 – 5)	0.043
**At the follow-up biopsy**			
Age (years)	12.2 ± 4.6	14.4 ± 4.6	0.31
Plasma creatinine (μmol/L)	54 (40 – 61)	87 (79 – 192)	<0.001
eGFR (mL/min/1.73m^2)	101 (94 – 123)	65 (28 – 76)	<0.001
dU-Prot[c] (g/day)	0.2 (0.1 – 0.3)	4.7 (3.3 – 8.2)	<0.001

ISKDC<br>I<br>II<br>IIIa<br>IIIb<br>IV<br>V	 1<br>15<br>2<br>3<br>0<br>0	 0<br>1<br>1<br>1<br>1<br>1	
SQC activity score	1 (1 – 2)	5 (4 – 6)	0.015
Decreased activity score from the diagnostic to follow-up biopsy, n (%)	16 (76%)	5 (100%)	0.55
SQC chronicity score	7 (4 – 8)	10 (9 – 11)	0.008
Increased chronicity score from the diagnostic to follow-up biopsy, n (%)	17 (81%)	5 (100%)	0.56
SQC tubulointerstitial score	3 (1 – 3)	3 (3 – 4)	0.062
Increased tubulointerstitial score from the diagnostic to follow-up biopsy, n (%)	16 (76%)	5 (100%)	0.55
MEST-C[b]<br>M1, n (%)<br>E1, n (%)<br>S1, n (%)<br>T1-2, n (%)<br>T>0%, n (%)<br>C1, n (%)<br>C2, n (%)<br>C1-2, n (%)<br>Total MEST-C score	 6 (32%)<br>1 (5%)<br>14 (74%)<br>1 (5%)<br>11 (58%)<br>2 (11%)<br>0<br>2 (11%)<br>1 (1 – 2)	 2 (40%)<br>2 (40%)<br>5 (100%)<br>2 (40%)<br>5 (100%)<br>3 (60%)<br>1 (20%)<br>4 (80%)<br>4 (3 – 4)	 >0.99<br>0.099<br>0.54<br>0.099<br>0.13<br>0.042<br>0.21<br>0.006<br>0.009
**At the last follow-up**			
Age (years)	18.7 ± 6.5	21.0 ± 7.8	0.61
Follow-up (years)	8.6 (6.8 – 10.5)	9.0 (5.8 – 13.2)	0.95
Plasma creatinine (μmol/L)	71 (57 – 75)	361 (158 – 450)	<0.001
eGFR (mL/min/1.73m^2)	88 (83 – 102)	14 (12 – 38)	0.001

Note. Statistical difference was tested with either Fisher's exact test or Mann Whitney U test.

eGFR, estimated glomerular filtration rate; dU-Prot, daily urine protein excretion; ISKDC, International Study of Kidney Disease in Children; SQC, semiquantitative classification; M, mesangial hypercellularity; E, endocapillary proliferation; S, segmental glomeruloscrelosis; T, tubular atrophy and/or interstitial fibrosis; C, crescents; NA, not applicable

[a]n=25

[b]n=24

[c]n=21
